# A Novel Locus for Ectodermal Dysplasia of Hair, Nail and Skin Pigmentation Anomalies Maps to Chromosome 18p11.32-p11.31

**DOI:** 10.1371/journal.pone.0129811

**Published:** 2015-06-26

**Authors:** Rabia Habib, Muhammad Ansar, Manuel Mattheisen, Muhammad Shahid, Ghazanfar Ali, Wasim Ahmad, Regina C. Betz

**Affiliations:** 1 Department of Biochemistry, Faculty of Biological Sciences, Quaid-i-Azam University, Islamabad, Pakistan; 2 Department of Genomic Mathematics, University of Bonn, Bonn, Germany; 3 Institute of Human Genetics, University of Bonn, Bonn, Germany; Università degli Studi di Milano, ITALY

## Abstract

Ectodermal dysplasias (EDs) are a large heterogeneous group of inherited disorders exhibiting abnormalities in ectodermally derived appendages such as hair, nails, teeth and sweat glands. EDs associated with reticulated pigmentation phenotype are rare entities for which the genetic basis and pathophysiology are not well characterized. The present study describes a five generation consanguineous Pakistani family segregating an autosomal recessive form of a novel type of ectodermal dysplasia. The affected members present with sparse and woolly hair, severe nail dystrophy and reticulate skin pigmentation. After exclusion of known gene loci related with other skin disorders, genome-wide linkage analysis was performed using Illumina HumanOmniExpress beadchip SNP arrays. We linked this form of ED to human chromosome 18p11.32-p11.31 flanked by the SNPs rs9284390 (0.113Mb) and rs4797100 (3.14 Mb). A maximum two-point LOD score of 3.3 was obtained with several markers along the disease interval. The linkage interval of 3.03 Mb encompassed seventeen functional genes. However, sequence analysis of all these genes did not discover any potentially disease causing-variants. The identification of this novel locus provides additional information regarding the mapping of a rare form of ED. Further research, such as the use of whole-genome sequencing, would be expected to reveal any pathogenic mutation within the disease locus.

## Introduction

Ectodermal dysplasias (EDs) are a large heterogeneous group of congenital disorders characterized by primary developmental defects in ectodermal appendages (hair, nails, teeth, sweat glands) during morphogenesis/development with or without accompanying defects in other tissues, organs and systems. So far, more than 200 EDs with different pathological and clinical manifestations have been reported but causative genes have been identified only in few ED phenotypes. The most reported sub-phenotype of ED is hypohidrotic ectodermal dysplasia (HED; MIM 305100) characterized by hypotrichosis, hypo- or anodontia (abnormal or absent teeth) and hypo-or anhidrosis (reduced or absent sweating) with disease-causing mutations in four genes, namely *EDA* (MIM 300451), *EDAR* (MIM 604095), *EDARADD* (MIM 606603) and *TRAF6* (MIM 602355). X-linked recessive (*EDA*), autosomal dominant and recessive (*EDAR*, *EDARADD*, *TRAF6*) inherited HED forms have been reported. Within the last years, many genetic causes of rare EDs were reported as e.g. mutations in the *HOXC13* gene (MIM 142976) [1], a member of the HOX family of transcription factors, causing pure hair and nail ectodermal dysplasia (PHNED; MIM 614931), mutations in the *KCTD1* gene (MIM 613420) [2] for scalp-ear-nipple syndrome (SENS; MIM 181270) and recently pathogenic variants in *GRHL2* gene causing an autosomal-recessive ectodermal dysplasia syndrome [3].

As reticulate pigmentation was one important feature of our newly collected family, we considered mainly genodermatoses involving skin pigmentation accompanied by diverse clinical conditions of different tissues and organ systems [4]. In few of these cases the presence of reticulate hyperpigmentation on different body parts including neck, shoulder, abdomen, proximal extremities and axillae associated with combination of defects in one or more ectodermal appendage(s) have been reported [5]. This includes conditions such as Naegeli-Franceschetti-Jadassohn syndrome (NFJS; MIM 161000), dermatopathia pigmentosa reticularis (DPR; MIM 125595), epidermolysis bullosa simplex with mottled pigmentation (EBS-MP; MIM 131960) and dyskeratosis congenita (DC). The two conditions, NFJS and DPR, are rare autosomal dominantly inherited allelic disorders with similar clinical manifestations. The hallmarks of core clinical features observed in these two conditions are absence of dermatoglyphics (fingerprints lines), reticulate hyperpigmentation of skin, palmoplantar keratoderma, abnormal sweating, nail dystrophy, dental anomalies and skin blistering [6,7]. The distinguishing features of lifelong presence of skin hyperpigmentation, partial alopecia, and absence of dental anomalies were observed in patients with DPR [8]. Heterozygous mutations in keratin 14 (*KRT14*; MIM 148066), located on chromosome 17q11.2-q21, are the underlying cause of NFJS and DPR syndromes [9,10]. Reticulate hyperpigmentation, nail dystrophy, leucoplakia, bone marrow failure and increased predisposition to malignancy are notable clinical features observed in patients with DC. The hyperpigmentation is manifested as brown-grey, and may be associated with guttate hypopigmentation, telangiectasia and atrophy with predilection sites being the neck, upper chest and upper arms. The DC is a genetically heterogeneous disorder with different modes of inheritance, although many cases result due to mutations in the X-linked gene dyskerin (*DKC1;* MIM 300126) [11].

The present study reports the clinical and molecular analysis of a consanguineous Pakistani kindred in which five males and one female were affected with a novel form of ED manifesting anomalies of hair, nails and skin pigmentation.

## Materials and Methods

### Human subjects

**Fig 1:**
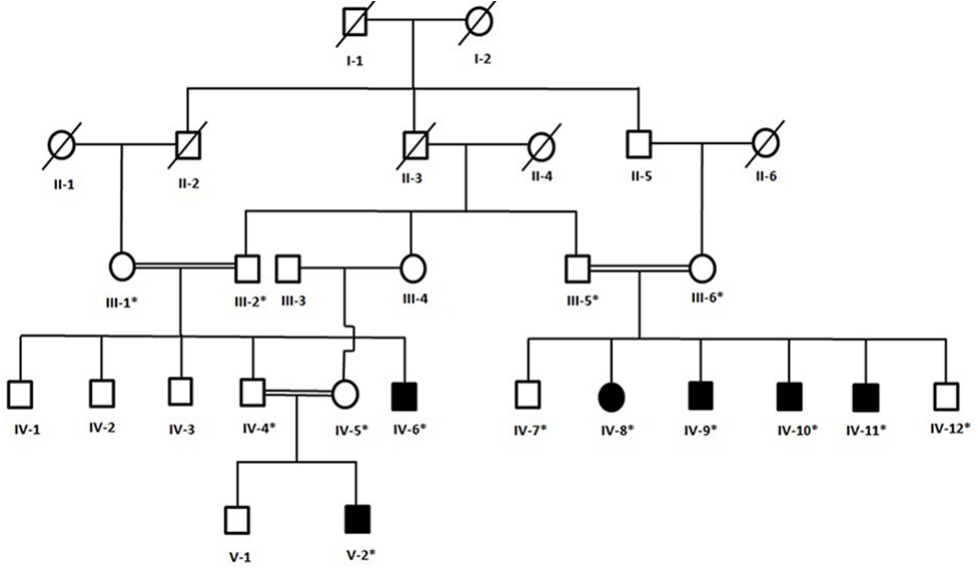
Pedigree of a consanguineous Pakistani family segregating an autosomal recessive form of a novel type of ectodermal dysplasia. Circles and squares represent females and males, respectively. Clear symbols represent unaffected individuals while filled symbols represent affected individuals. Symbols with asterisk represent DNA samples available for the molecular analysis.

A five generation consanguineous family demonstrating an unreported form of ectodermal dysplasia involving anomalies of hair, nail and skin pigmentation was ascertained from a remote region of Pakistan. The pedigree provided convincing evidence of an autosomal recessive mode of inheritance of the phenotype (Fig 1). Affected members of the family underwent careful clinical examination at a local government hospital. The study was approved by the Institutional Review Board (IRB) of Quaid-i-Azam University, Islamabad Pakistan. Informed written consent for the study including presentation of photographs for publication was obtained from all participating subjects and their guardians. The consent procedure including use of form was approved by IRB Quaid-i-Azam University Islamabad Pakistan.

### Extraction of DNA and genotyping

Fourteen individuals including six affected (IV-6, IV-8, IV-9, IV-10, IV-11, V-2) and eight unaffected members (III-1, III-2, III-5, III-6, IV-4, IV-5, IV-7, IV-12) of the family provided blood samples for DNA-based analysis. Genomic DNA from the blood samples was extracted using GenElute Blood Genomic DNA Kit (Sigma-Aldrich, St. Louis, MO, USA). PCR amplification using microsatellite markers was performed as described earlier [12].

### Exclusion mapping

Linkage in the present family was first tested using microsatellite markers tightly linked to known genes involved in different forms of ectodermal dysplasia phenotypes. These included Loricrin (*LOR*; MIM 152445) on chromosome 1q21.3 ([chr1:153232179–153234600] D1S2858, D1S1595, D1S2624, D1S1653), Plakophilin (*PKP1;* MIM 601975) on 1q32.1 ([1:201,252,579–201,302,120 ] D1S1678, D1S1725, D1S456, D1S2156), *EDARADD* (MIM 606603) on 1q43 ([1:236,557,679–236,648,007] D1S235, D1S2850, D1S517, D1S1149), *EDAR* (MIM 604095) on 2q12.3([2:109,510,926–109,605,827] D2S1889, D2S1893, D2S1891, D2S1888), *HPGD* (MIM 601688) on 4q32.3-q43.2 ([4:175,411,327–175,444,048]D4S2388, D4S1597, D4S621, D4S1539, D4S3030), Corneodesmosin (*CDSN;* MM 602593) on 6p21.33 ([6:31,082,864–31,088,251] D6S265, D6S952, D6S273, D6S1666), Frizzled 6 (*FZD6;* MIM 603409) on 8q22.3([8:104,310,660–104,345,093 ]GAAT1A4, D8S559, D8S1762, D8S1714, D8S545), Ectodermal dysplasia of hair-nail locus on 10q24.32-q25.1 (MIM 614927; [10:103,000,000–111,900,000]D10S1710, D10S1267, D10S1264, D10S254, D10S1741), Poliovirus Receptor-Like 1 (*PVRL1;* MIM 600644) on 11q23.3 ([11:119,508,807–119,599,434] D11S1999, D11S4189, D11S1334, D11S1307), Suppression of tumorigenicity (*ST14;* MIM 606797) at 11q24.3 ([11:130,029,681–130,080,256] D11S569, D11S926, D11S1307, D11S861), Type II Keratin (*KRT8;* MIM 602153, *KRT83;MIM* 602765, *KRT85;* MIM 602767, *KRT86;* MIM 601928) on 12p11.1-q21.1([12:33,300,001–75,700,000]D12S270, D12S1604, D12S103, D12S312, D12S1724, D12S1298, D12S1022), Gap Junction Proteins (*GJB;* MIM 604418, *GJB2;* MIM 121011) on 13q12.11 ([13:19,500,001–23,300,000] D13S175, D13S633, D13S115, D13S1275, D13S292), Transglutaminase 1 (*TGM1;* MIM 190195) on 14q12 ([14:24,718,158–24,733,114] D14S1430, D14S581, D14S64, D14S1280), Isolated Congenital Nail Dysplasia (ICND; MIM 605779) on 17p13 ([17:0–10,700,000] D17S926, D17S1840, D17S1529, D17S1528, D17S1845), Type I Keratin (*KRT14*; MIM 148066), Naegeli-Franceschetti-Jadassohn Syndrome and Dermatopathia Pigmentosa Reticularis locus on 17q12-q21.2([17:31,800,000–39,743,146] D17S1814, D17S1299, D17S800, D17S1787, D17S1860), Envoplakin (*EVPL;* MIM 601590) on 17q25.1-q25.3 ([17:74,002,925–74,023,532] D17S939, D17S2195, D17S674, D17S1830, D17S914), Transglutaminase 3 (*TGM3;* MIM 600238) and R-Spondin 4 (*RSPO4;* MIM 610573) on 20p13 ([20:1–5,100,000] D20S103, D20S117, D20S199, D20S906), and Transglutaminase 2 (*TGM2;*MIM 190196) on 20q11.23 ([20:36,756,862–36,794,909] D20S847, D20S834, D20S881, D20S908).

### Human genome scan

Human genome scan was performed using HumanOmniExpress beadchip arrays of Illumina Infinium HD Assay (Illumina Inc., San Diego, CA, USA) containing more than 700,000 SNP loci with an average mean distance of 4 kb. The Infinium HD Assay genotyping reaction steps were performed according to the manufacturer’s specifications (Illumina Inc., San Diego, CA, USA) at the Department of Genomics, Life and Brain Center, University of Bonn, Germany. The SNP genotypes generated from normalized bead intensity data for each sample were analyzed with Illumina Genome Studio v1.0.2 software using manufacturer’s default cluster settings, combined with the normalized measure of the total signal intensity for the two alleles of a SNP defined as the Log R ratio (LRR) and the normalized measure of the allelic intensity ratio of the two alleles defined as the B allele frequencies (BAF), which were used to detect homozygous regions and other chromosomal anomalies including deletion and duplication on megabase pair scale. Due to the autosomal recessive mode of inheritance of the clinical phenotype in the family, homozygous regions were also determined by HomozygosityMapper [13] (http://www.homozygositymapper.org/). Mendelian inconsistencies were evaluated using PEDCHECK [14] and MERLIN [15]. Two-point linkage analysis was carried out using MLINK program of FASTLINK computer package [16].

### Candidate gene screening

At the time of the study seventeen genes, identified in the linked region on chromosome 18, were prioritized for sequencing on the basis of information pertaining to their expression in skin and signaling pathways from the University of California Santa Cruz (UCSC) Human Genome Browser (http://genome.ucsc.edu/cgi-bin/hgGateway) and Genedistiller [17]. Subsequently exons, splice junction sites, 5’untranslated regions (UTR) and polyadenylation sites in 3’ UTR of all 17 genes were screened for potential sequence variants in two affected (IV-8, IV-10) and one unaffected (III-5) individual of the family. Primer sequences for PCR-amplification of the genes were designed from their intronic sequences by Primers3 version 0.4.0 software [18] (http://frodo.wi.mit.edu/primer3/) using the reference sequences obtained from UCSC Human Genome browser and Ensemble Genome Browser (http://www.ensemble.org), and checked for specificity using basic local alignment search tool (BLAST; http://www.ncbi.nlm.nih.gov/blast). The amplified PCR products were purified under standard conditions as described earlier by Habib *et al*.[12] and sequence analysis was carried out with the ABI PRISM Big Dye Terminator Cycle Sequencing V3.1 Ready Reaction Kit. The sequencing products were purified by PrepEase Sequencing Dye Clean-Up Kit (USB Corporation, Ohio, USA) in accordance with manufacture’s protocol. The purified products were subjected to denaturing electrophoresis loaded on the ABI PRISM 3100 Genetic Analyzer (Applera Corp., Foster City, CA USA). The sequence data was aligned with control sequence via BIOEDIT sequence alignment editor version 6.0.7 (http://www.mbio.ncsu.edu/BioEdit/bioedit.html).

## Results

### Clinical findings

An uncharacterized form of ectodermal dysplasia was observed in six individuals (IV-6, IV-8, IV-9, IV-10, IV-11, V-2) of the family presented here (Fig 1). The affected members were between 12 to 28 years of age at the time of the study. All the affected individuals were the product of consanguineous unions and born at full term of normal pregnancies. Detailed clinical evaluation performed by medical officers at a local government hospital revealed the under-mentioned clinical features in the affected subjects of the family (Table 1).

**Table 1:** Clinical features of ED syndrome observed in affected members of family.

Organ/Appendage	Clinical Features
**Skin**	***Reticulate hyperpigmentation***
	Presence of net like dark brown gray patches associated with guttate hypopigmentation
	Localized on neck, abdomen, chest, axillae and extremities
	Age of Onset: early adolescence
	Lifelong Presence after appearance
**Hair**	***Alopecia***
	Sparse, thin scalp hair
	Hypopigmentation, woolly texture of scalp hair
	Sparse eyebrows/ eyelashes
	Sparse beard, mustache hair in affected males
	Absence of body hairs (chest, arms, axillae)
**Nail**	***Nail dystrophy***
	Misshaped pigmented finger- and toenails
	Micronychia
	Thickened curved nail plate (oncochauxis)
	Transverse melanonychia (dark coloured spots on nail plate) on finger- and toenails
	Onycholysis

#### Nails

**Fig 2:**
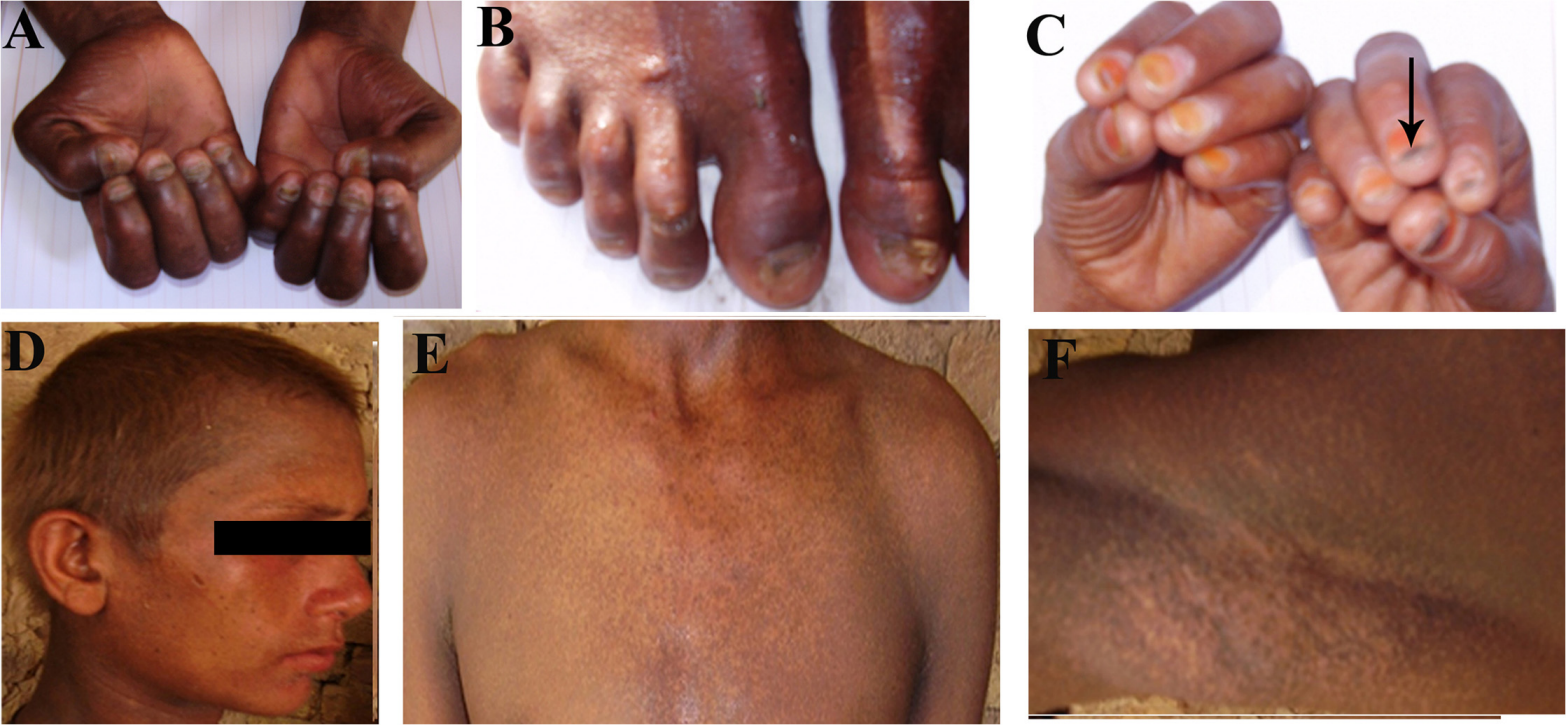
Clinical features of affected members IV-9 at 15 years and IV-11 at 21 years of age. Finger- and toenails of both hands and feet of patient (IV-11) showing nail dystrophy (A, B). Arrow points towards transverse melanonychia on fingernail of patient (IV-9) (C). Thin hypopigmented scalp hairs and sparse eyelashes in patient IV-9 (D). The Patient IV-11 displays reticulate pattern of cutaneous hyperpigmentation on neck, chest (E) and underarm area (F).

At the time of birth, nail growth was normal. Dystrophy of the nails appeared during second decade of the patient’s life. Severe onychodystrophy manifested as dystrophic misshaped pigmented finger- and toenails in all six affected members of the family. Other features associated with nail dystrophy noted in the patients included micronychia (small nails), oncochauxis (thickened curved nail plate), transverse melanonychia (dark colored spots on nail plate) and onycholysis (detachment of the nail from nail bed) (Fig 2A, 2B and 2C). All the patients reported occurrence of pain during physical work.

#### Hairs

Sparse thin scalp hair of woolly texture, and sparse eyebrows and eyelashes were observed in all the affected members. In affected males beard and moustache hair were sparse as well. Hair was missing on other parts of the body (Fig 2D).

#### Skin

Reticulate pattern of hyperpigmentation manifested in form of net like dark brown gray patches observed on different parts of the body including neck, abdomen, chest, axillae, and extremities. Associated condition of guttate hypopigmentation was noted on some of the body parts. Information provided by the family members revealed that the reticulate hyperpigmentation develops during early adolescence in the patients (Fig 2E and 2F) and after appearance stays on permanent basis.

Sweating and dentition was found normal in all patients. No underlying defects in renal, liver and other organ systems were reported. Immunological, systemic involvement and predisposition to malignancy was not reported in the patients. Medical history of the patients showed no exposure to drugs and chemical solvents that could have induced hyperpigmentation in affected individuals. The patients followed normal pattern of growth development. Biological specimen for skin biopsy was not available due to non-availability of consent of the affected individuals.

### Mapping Ectodermal Dysplasia on chromosome 18p11.32-p11.31

After excluding linkage in the family to genes involved in skin related disorders (*LOR*, *PKP1*, *EDARADD*, *EDAR*, *HPGD*, *CDSN*, *FZD6*, *PVRL1*, *ST14*, *GJB*, *GJB2*, *TGM1*, *KRT14*, *EVPL*, *TGM2*, *TGM3*, *RSPO4*, NFJ and DPR loci, Type II Keratin gene cluster), the present family was subjected to a genome-wide scan. The whole genome scan was performed using HumanOmniExpress beadchip arrays of Illumina Infinium HD Assay (Illumina Inc., San Diego, CA, USA) on fourteen DNA samples including six affected (IV-6, IV-8, IV-9, IV-10, IV-11, VII-7) and eight unaffected individuals (III-1, III-2, III-5, III-6, IV-4, IV-5, IV-7, IV-12) of the present family. A single run of homozygous SNP markers spanning about 3.03 Mb flanked by SNPs rs9284390 (0.113 Mb) and rs4797100 (3.14 Mb) on chromosome 18p11.32-p11.31 was detected. The physical positions of the SNP markers are according to dbSNP132 (http://genome.ucsc.edu/cgi-bin/hgGateway). Analysis of the genome scan genotypes within this region by PEDCHECK and MERLIN did not elucidate any genotyping errors. The results of the two-point linkage analysis using MLINK program of FASTLINK computer package [16] are shown in Table 2. The highest two-point LOD score at zero recombination fraction (θ = 0.00) of 3.33 was obtained with multiple SNP markers, supporting linkage to this region.

**Table 2:** Two-point LOD score between ED syndrome and SNP markers on chromosome 18p11.32-p11.31.

Marker Information		Two-point Lod Score at Recombination fraction θ ^b^ =						
SNP ID	Marker Position (bp) ^a^	0	0.01	0.05	0.1	0.2	0.3	0.4
**rs9284390**	**112535**	**3.3391**	**3.2673**	**2.9765**	**2.6062**	**1.8523**	**1.099**	**0.3901**
**rs7234528**	**136305**	**3.3391**	**3.2673**	**2.9765**	**2.6062**	**1.8523**	**1.099**	**0.3901**
**rs4797697**	**139767**	**3.3391**	**3.2673**	**2.9765**	**2.6062**	**1.8523**	**1.099**	**0.3901**
**rs551835**	**160887**	**3.3391**	**3.2673**	**2.9765**	**2.6062**	**1.8523**	**1.099**	**0.3901**
**rs9945820**	**373670**	**3.3391**	**3.2673**	**2.9765**	**2.6062**	**1.8523**	**1.099**	**0.3901**
**rs3932728**	**398147**	**3.3391**	**3.2673**	**2.9765**	**2.6062**	**1.8523**	**1.099**	**0.3901**
**rs11662321**	**404000**	**3.3391**	**3.2673**	**2.9765**	**2.6062**	**1.8523**	**1.099**	**0.3901**
**rs4798145**	**407958**	**3.3391**	**3.2673**	**2.9765**	**2.6062**	**1.8523**	**1.099**	**0.3901**
**rs551835**	**160887**	**3.3391**	**3.2673**	**2.9765**	**2.6062**	**1.8523**	**1.099**	**0.3901**
**rs9945820**	**373670**	**3.3391**	**3.2673**	**2.9765**	**2.6062**	**1.8523**	**1.099**	**0.3901**
**rs3932728**	**398147**	**3.3391**	**3.2673**	**2.9765**	**2.6062**	**1.8523**	**1.099**	**0.3901**
**rs11662321**	**404000**	**3.3391**	**3.2673**	**2.9765**	**2.6062**	**1.8523**	**1.099**	**0.3901**
**rs4798145**	**407958**	**3.3391**	**3.2673**	**2.9765**	**2.6062**	**1.8523**	**1.099**	**0.3901**
**rs4798418**	**612753**	**3.3391**	**3.2673**	**2.9765**	**2.6062**	**1.8523**	**1.099**	**0.3901**
**rs505140**	**624631**	**3.3391**	**3.2673**	**2.9765**	**2.6062**	**1.8523**	**1.099**	**0.3901**
**rs11877057**	**648465**	**3.3391**	**3.2673**	**2.9765**	**2.6062**	**1.8523**	**1.099**	**0.3901**
**rs9966612**	**649311**	**3.3391**	**3.2673**	**2.9765**	**2.6062**	**1.8523**	**1.099**	**0.3901**
**rs12456880**	**846744**	**3.3391**	**3.2673**	**2.9765**	**2.6062**	**1.8523**	**1.099**	**0.3901**
**rs4797325**	**889905**	**3.3391**	**3.2673**	**2.9765**	**2.6062**	**1.8523**	**1.099**	**0.3901**
**rs17500692**	**900789**	**3.3391**	**3.2673**	**2.9765**	**2.6062**	**1.8523**	**1.099**	**0.3901**
**rs1893154**	**905125**	**3.3391**	**3.2673**	**2.9765**	**2.6062**	**1.8523**	**1.099**	**0.3901**
**rs1010003**	**965990**	**3.3391**	**3.2673**	**2.9765**	**2.6062**	**1.8523**	**1.099**	**0.3901**
**rs789063**	**1006470**	**3.3391**	**3.2673**	**2.9765**	**2.6062**	**1.8523**	**1.099**	**0.3901**
**rs987611**	**1306068**	**3.3391**	**3.2673**	**2.9765**	**2.6062**	**1.8523**	**1.099**	**0.3901**
**rs12604976**	**1604960**	**3.3391**	**3.2673**	**2.9765**	**2.6062**	**1.8523**	**1.099**	**0.3901**
**rs2580128**	**1804897**	**3.3391**	**3.2673**	**2.9765**	**2.6062**	**1.8523**	**1.099**	**0.3901**
**rs1595674**	**1817362**	**3.3391**	**3.2673**	**2.9765**	**2.6062**	**1.8523**	**1.099**	**0.3901**
**rs8085513**	**1997528**	**3.3391**	**3.2673**	**2.9765**	**2.6062**	**1.8523**	**1.099**	**0.3901**
**rs16942467**	**2033590**	**3.3391**	**3.2673**	**2.9765**	**2.6062**	**1.8523**	**1.099**	**0.3901**
**rs491222**	**2089892**	**3.3391**	**3.2673**	**2.9765**	**2.6062**	**1.8523**	**1.099**	**0.3901**
**rs566821**	**2104793**	**3.3391**	**3.2673**	**2.9765**	**2.6062**	**1.8523**	**1.099**	**0.3901**
**rs476941**	**2111078**	**2.7828**	**2.7241**	**2.4868**	**2.1856**	**1.57**	**0.9432**	**0.346**
**rs8086568**	**2217798**	**3.3391**	**3.2673**	**2.9765**	**2.6062**	**1.8523**	**1.099**	**0.3901**
**rs627202**	**2292491**	**3.3391**	**3.2673**	**2.9765**	**2.6062**	**1.8523**	**1.099**	**0.3901**
**rs7230412**	**2483514**	**3.3391**	**3.2673**	**2.9765**	**2.6062**	**1.8523**	**1.099**	**0.3901**
**rs2677906**	**2491875**	**3.3391**	**3.2673**	**2.9765**	**2.6062**	**1.8523**	**1.099**	**0.3901**
**rs2682136**	**2492934**	**3.3391**	**3.2673**	**2.9765**	**2.6062**	**1.8523**	**1.099**	**0.3901**
**rs7227757**	**2641035**	**3.3391**	**3.2673**	**2.9765**	**2.6062**	**1.8523**	**1.099**	**0.3901**
**rs9947736**	**2641753**	**3.3391**	**3.2673**	**2.9765**	**2.6062**	**1.8523**	**1.099**	**0.3901**
**rs1893123**	**2733990**	**3.3391**	**3.2673**	**2.9765**	**2.6062**	**1.8523**	**1.099**	**0.3901**
**rs559994**	**2804129**	**3.3391**	**3.2673**	**2.9765**	**2.6062**	**1.8523**	**1.099**	**0.3901**
**rs673783**	**2825418**	**3.3391**	**3.2673**	**2.9765**	**2.6062**	**1.8523**	**1.099**	**0.3901**
**rs11664521**	**2831496**	**3.3391**	**3.2673**	**2.9765**	**2.6062**	**1.8523**	**1.099**	**0.3901**
**rs583523**	**2874156**	**3.3391**	**3.2673**	**2.9765**	**2.6062**	**1.8523**	**1.099**	**0.3901**
**rs637647**	**2881839**	**3.3391**	**3.2673**	**2.9765**	**2.6062**	**1.8523**	**1.099**	**0.3901**
**rs1164**	**2917152**	**3.3391**	**3.2673**	**2.9765**	**2.6062**	**1.8523**	**1.099**	**0.3901**
**rs7232826**	**3031067**	**0.008**	**1.6966**	**2.0825**	**1.9927**	**1.5044**	**0.9037**	**0.3114**
**rs948299**	**3087943**	**3.3391**	**3.2673**	**2.9765**	**2.6062**	**1.8523**	**1.099**	**0.3901**
**rs8098020**	**3093474**	**3.3391**	**3.2673**	**2.9765**	**2.6062**	**1.8523**	**1.099**	**0.3901**
**rs4798067**	**3099681**	**0.009**	**1.7956**	**2.0745**	**2.0927**	**1.7134**	**0.9737**	**0.3114**
rs4797100	3138553	-2.8987	-0.8017	-0.2265	-0.0677	-0.0068	-0.0043	-0.0008
rs6506070	3141689	-0.7508	0.9879	**1.449**	**1.4505**	**1.1242**	0.6374	0.1308
rs9960004	3152450	-0.7508	0.9879	**1.449**	**1.4505**	**1.1242**	0.6374	0.1308
rs6506073	3156061	-3.187	-1.1101	-0.4793	-0.2583	-0.1066	-0.0588	-0.0328
rs9952207	3162390	-3.0128	-0.9634	-0.3638	-0.1754	-0.0642	-0.0284	-0.008
rs4798069	3163771	-0.7508	0.9879	**1.449**	**1.4505**	**1.1242**	0.6374	0.1308
rs10153410	3163965	-0.7508	0.9879	**1.449**	**1.4505**	**1.1242**	0.6374	0.1308

**a** Physical positions of the SNPs are according to the dbSNP132 (http://genome.ucsc.edu/cgi-bin/hgGateway)

**b** Recombination fraction.

### Mutation analysis of candidate genes

**Fig 3:**
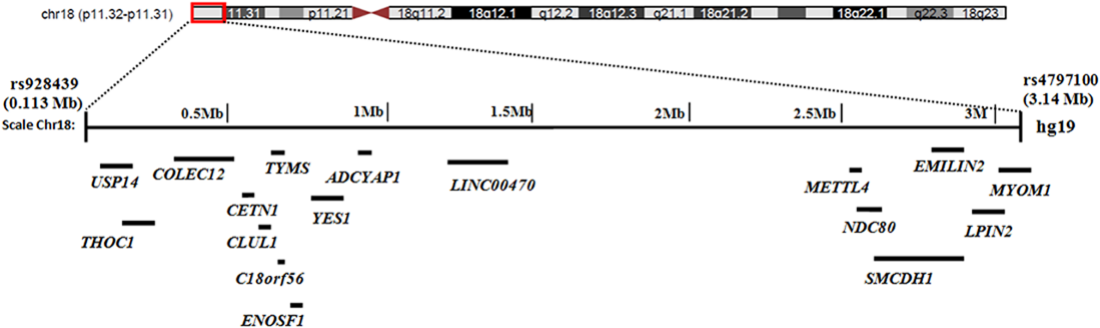
Ideogram of chromosome 18 displaying positions of the functional genes along the linkage interval (Chr18p11.32-p11.31 on GRCH37/hg19 assembly) of 3.03 Mb identified in the family.

According to Genedistiller [17], the candidate region of 3.03 Mb on chromosome 18p, identified in the present family, contained 16 protein coding and one long intergenic non-protein coding RNA genes [*ADCYAP1* (MIM 102980), *C18orf56* (Entrez Gene: 494514), *CETN1* (MIM 603187), *CLUL1* (Entrez Gene: 27098), *COLEC12* (MIM 607621), *EMILIN2* (MIM 608928), *ENOSF1* (MIM 607427), *LPIN2* (MIM 605519), *METTL4* (MIM 612472), *MYOM1* (MIM 603508), *NDC80* (MIM 607272), *SMCDH1* (MIM 614982), *THOC1* (MIM 606930), *TYMS* (MIM 188350), *USP14* (MIM 607274), *YES1* (MIM 164880) and *LINC00470* (Entrez Gene: 56651)]. Positions of the functional genes along the linkage interval on chromosome 18p are illustrated in Fig 3 for more clarity. Major functions assigned to the fourteen genes include pyrimidine biosynthesis (*TYMS*, *ENOSF1*)proteasome associated ubiquitin recycling (*USP14*), cell signaling component (*ADCYAP1*, *YES1*), innate immune responses (*COLEC12*), role in mitosis (*CETN1*, *NDC80*), spliced transcript processing and transport (*THOC1*), chromatin modification (*SMCDH1*, *METTL4*) muscle structural component (*MYOM1*), vessel assembly regulation (*EMILIN2*) and adipose tissue formation (*LPIN2*). The function of three genes (*C18orf56*, *CLUL1*, *LINC00470*) were unknown. None of these genes had been reported to cause any type of Ectodermal dysplasia. Although functionally significant, but none of these genes were considered as promising candidate for the disorder involving pigmentation and ectodermal appendages anomaly in the linkage interval. Therefore, all the genes were screened for the sequence variants. The genes were sequenced in two affected (IV-8, IV-10) and one unaffected (III-5) individual of the family. Sequence analysis with standard sequence of the exons, intron-exons boundaries, 5’ UTR regions and polyadenylation site in 3’ UTR of all 17 genes failed to detect any functional sequence variant which could be responsible for causing the disease phenotype observed in the family. The disease interval was also assessed for the presence of any potential deletion or duplication using Genome Studio v1.0.2 software. No deletion, duplication or chromosomal aberration was detected.

## Discussion

In the present study, we have presented a clinical and molecular analysis of an unreported ED condition observed in a five generation consanguineous family of Pakistani origin. All six affected individuals of the family exhibited clinical features including hypotrichosis, nail dysplasia and reticulate hyperpigmentation. The later condition was found restricted to certain body parts including neck, abdomen, extremities and axillae. An extensive literature review revealed that some of the clinical features observed in affected members of the family were reported earlier in at least three other forms of ED including NFJS, DPR and DC. However, absence of clinical features such as palmoplantar keratoderma, dental anomalies, skin blistering, multiple small punctate keratoses of palms and soles, and hypohidrosis in the present family distinguishes the condition from NFJS and DPR. In case of NFJS, the reticulate hyperpigmentation tend to fade with age while in DPR pigmentation anomaly shows lifelong presence and onset occurs as early as birth to 2 years of age [6–8]. On the other hand in the ED phenotype observed in the family reported here, the reticulate hyperpigmentation appears during early adolescence and then stays as such for the rest of life. Similarly, the ectodermal disorder demonstrated in the current family can be distinguished from multisystemic pigmentary disorder DC by absence of associated phenotypes including mucosal leukoplakia, development of immune deficiency, pulmonary complications, malignancy, bone marrow failure and various other somatic abnormalities. In DC, reticular skin pigmentation and nail dystrophy are associated with diverse clinical findings affecting other organs and organ system [11]. Therefore, combination of the clinical findings such as hypotrichosis, nail dystrophy, and reticulate hyperpigmentation without association of any other underlying defects in organs and organ systems convincingly supported the demonstration of a novel type of entity of genodermatosis in the consanguineous family reported in present study.

As expected, genotyping using microsatellite markers excluded linkage in the family to several genes involved in causing ectodermal dysplasias. The human genome was then scanned using Illumina HumanOmniExpress SNPs beadchip microarray. Analysis of the genome scan results revealed linkage of the family to chromosome 18p11.32-p11.31 with a significant LOD score value (Table 2). The 3.03 Mb homozygous region, found in affected individuals of the family, encompassed 16 protein encoding and one non-protein encoding RNA genes (Fig 3). Since, none of these genes were reported to be involved in the disorders of ectodermal appendages therefore all seventeen genes had been examined. Sequence analysis, however, failed to detect potential disease causing variants. We had also looked for the presence of deletions, duplications and other chromosomal aberrations in the linked region using Illumina genome studio v1.0.2 software. The software is handicapped with its limitations of not detecting deletions/duplications below 50 kb size and as such was unable to provide information about any minor chromosomal aberrations. Still, the possibility of any disease causing sequence variant exists in regulatory regions of the genes or small deletions/insertions/duplications in the linkage interval cannot be completely ruled out. Mapping of the linkage interval on chromosome 18p11.32-p11.31 and mutation screening of the genes for potential sequence variant is the first step towards deciphering the molecular basis of this congenital disorder. Targeted next generation sequencing (NGS) of this region might be helpful in identifying disease causing sequence variant or other possible genetic aberration that is usually impossible to detect by conventional Sanger sequencing. The possible disease causative mutation could be a variant in any cis-regulatory region of the genes or a small structural variant such as copy number variant (CNV) of <50Kb size in intergenic regions. According to recent updates on UCSC genome browser in the latest human assembly GRCh38/hg38, the linkage interval on chromosome 18p11.32-p11.31 shows annotation of eleven small ncRNA genes (six piwi interacting RNAs [piRNAs], two small cytoplasmic RNAs [scRNAs] and one microRNA [miRNA]) of uncharacterized functions. Mutation causing abnormality of the function in any of these unknown ncRNA cannot be ruled out as probable cause for this disorder. Eventual identification of a gene or any other DNA variant responsible for this novel form of ectodermal dysplasia at this locus will enhance our knowledge about the molecular mechanisms involved in the development/maintenance of the tissues affected in the present family.
